# Granulation of Bismuth Oxide by Alginate for Efficient Removal of Iodide in Water

**DOI:** 10.3390/ijms232012225

**Published:** 2022-10-13

**Authors:** Tae-Hyun Kim, Chihyun Seo, Jaeyoung Seon, Anujin Battulga, Yuhoon Hwang

**Affiliations:** 1 Department of Environmental Engineering, Seoul National University of Science and Technology, Seoul 01811, Korea; 2 Water Quality Center, Chemicals & Environment Research Institute, Korea Testing & Research Institute, Gyeonggi-do 13810, Korea

**Keywords:** bismuth oxide, alginate, granulation, iodide adsorption

## Abstract

The granulation of bismuth oxide (BO) by alginate (Alg) and the iodide adsorption efficacy of Alg–BO for different initial iodide concentrations and contact time values were examined. The optimal conditions for Alg–BO granulation were identified by controlling the weight ratio between Alg and BO. According to the batch iodide adsorption experiment, the Alg:BO weight ratio of 1:20 was appropriate, as it yielded a uniform spherical shape. According to iodide adsorption isotherm experiments and isotherm model fitting, the maximum sorption capacity (*q*_m_) was calculated to be 111.8 mg/g based on the Langmuir isotherm, and this value did not plateau even at an initial iodide concentration of 1000 mg/L. Furthermore, iodide adsorption by Alg–BO occurred as monolayer adsorption by the chemical interaction and precipitation between bismuth and iodide, followed by physical multilayer adsorption at a very high concentration of iodide in solution. The iodide adsorption over time was fitted using the intraparticle diffusion model. The results indicated that iodide adsorption was proceeded by boundary layer diffusion during 480 min and reached the plateau from 1440 min to 5760 min by intraparticle diffusion. According to the images obtained using cross-section scanning electron microscopy assisted by energy-dispersive spectroscopy, the adsorbed iodide interacted with the BO in Alg–BO through Bi–O–I complexation. This research shows that Alg–BO is a promising iodide adsorbent owing to its high adsorption capacity, stability, convenience, and ability to prevent secondary pollution.

## 1. Introduction

At present, nuclear energy is being widely used as a reliable and clean energy source for electricity. Despite those advantages, nuclear accidents, such as those of the Chernobyl and Fukushima nuclear power plants, may release dangerous radioisotopes such as Cs-134, Cs-137, I-129, and I-131 into the environment [1,2]. The recovery of radioactive iodine (I-129 and I-131) from the environments is challenging owing to its high radioactive toxicity, solubility, and mobility [3,4]. Moreover, the considerably longer half-life of I-129 (1.57 ± 0.006 × 10^7^ years) than that of I-131 (8 days) means that it may expose humans and other living organisms to chronic toxicity [5,6]. In aqueous systems, iodine (I_2_) mainly occurs as iodide (I^−^) and iodate (IO_3_^−^), depending on the pH, with iodide being the dominant species at neutral pH [7,8,9]. The release of radioactive iodide is attributable to the dissolution of CsI, which is typically used as nuclear fuel in light-water reactors [10]. The iodine is mainly released as a gaseous contaminant due to its high volatility, but it can be easily dissolved in the resulting water form of radioactive iodide ions. Therefore, effective strategies to remove radioactive iodide from the aqueous medium must be established to ensure the safety of the nuclear industry and environment.

Many researchers have examined iodide adsorption with mineral-based, metal-based, polymer-based, and carbon-based adsorbents in aqueous systems [11,12,13,14,15,16]. Bismuth-based adsorbents, e.g., bismuth oxide (BO), basic bismuth nitrate, and bismuth subcarbonate, have attracted considerable research interest because of their high selectivity for iodide and low toxicity and cost [17,18,19]. The high iodide selectivity of bismuth-based adsorbents is attributable to the formation of effective Bi–O–I compounds as a novel waste form [20]. It was reported that ~76% of the removal capacity was still maintained in the presence of chloride when iodide adsorption on mesoporous bismuth oxide was performed with 6 mM chloride ions [17]. The stable adsorption behavior under a wide pH range of 4–11 was also verified using Microrosette-like δ-Bi_2_O_3_ [21]. However, powdered adsorbents are challenging to separate and recycle. Granulation of the powdered form of particulate matter was considered an effective process for enhancing its practical applicability [22,23,24,25]. Many researchers have attempted to prepare granules of adsorbents through pellet molding methods by applying strong compressive force as a facile methodology. Notably, the equipment for pellet molding is expensive, and the process requires the application of high pressure, which may block the pores on the surface or change the internal structure of the adsorbents [26]. To address these problems, bismuth-based adsorbents were fixed on a substance or granulated using polymers [27]. Among various polymers, alginate (Alg) extracted from brown algae is a promising candidate owing to its environmental friendliness, low cost, and easy preparation method by simply dropping it on a crosslinking agent such as Ca^2+^ ions [28]. Owing to the ionotropic gelation of spherical drops, the polyguluronate units in the alginate molecules form a chelated structure with metal ions. Then, the chelate structure is transformed to become kinetically stable toward dissociation while the polymannuronate units show normal cations binding [29]. According to the two interactions, granulation by Alg leads to the formation of spherical-shaped beads [30]. Spherical Alg granules are convenient to use as adsorbents in practical applications. They can help avoid secondary pollution resulting from dissolution, as reported in several articles with various powder adsorbents, such as iron oxide, clay, and activated carbon [31,32,33].

Considering these aspects, in this study, BO was prepared using the solvothermal method and then granulated with Alg to realize iodide adsorption in an aqueous system. Specifically, Alg–BO was prepared in a facile manner by dropping the Alg and BO suspension into a calcium chloride (CaCl_2_) solution to achieve a uniform spherical shape. The granulation of BO by Alg was characterized by powder X-ray diffraction (PXRD), Fourier-transform infrared (FT-IR), Brunauer–Emmett–Teller (BET) surface area analysis, and microscopic analysis. The optimal conditions for granulation were identified by controlling the weight ratio of Alg to BO through batch iodide adsorption experiments. Moreover, iodide adsorption isotherm experiments were conducted, and the results were fitted using the Langmuir and Freundlich models for the adsorption isotherm. According to the kinetic experiment results fitted with the intraparticle diffusion model, iodide adsorption occurred through boundary layer diffusion in the initial stage and then through intraparticle diffusion. The effect of pH on iodide adsorption, as well as the integrity of granules, was also investigated. After iodide adsorption, the Alg–BO sample was characterized via PXRD, FT-IR, and scanning electron microscopy (SEM) assisted by energy-dispersive spectroscopy (EDS) to evaluate the structural changes and adsorbed iodide distribution.

## 2. Results and Discussion

### 2.1. Optimization of Alg–BO Preparation Conditions for Iodide Adsorption

The granulation conditions were optimized by preparing Alg–BO considering five weight ratios of Alg to BO (1:5, 1:10, 1:20, 1:30, and 1:40). As shown in Figure S1, the beads prepared with weight ratios ranging from 1:5 and 1:20 were spherical with a diameter of approximately 0.3 mm. When the ratio was increased to 1:30 and 1:40, the shape of Alg–BO was slightly elongated and irregular. At higher BO ratios, the Alg was inadequate to establish a stable structure once it reacted with the calcium ions in the bath. Similar results have been observed for halloysite–alginate and organoclay–alginate nanocomposites [33,34]. The sphericity of grains of the filtration bed applied for water treatment was considered an important parameter for column design as it affects the bed porosity. Siwiec (2007) reported that high sphericity could bring lower porosity in the filter bed [35]; therefore, a denser adsorbent bed could be expected. Furthermore, as adsorption performance is affected by the adsorbent mass in the unit bed volume, the bed filled with spherical granules could expect a higher performance and lifetime. These results indicated that Alg:BO ratios of 1:5 to 1:20 were suitable for stable bead formation. 

**Figure 1:**
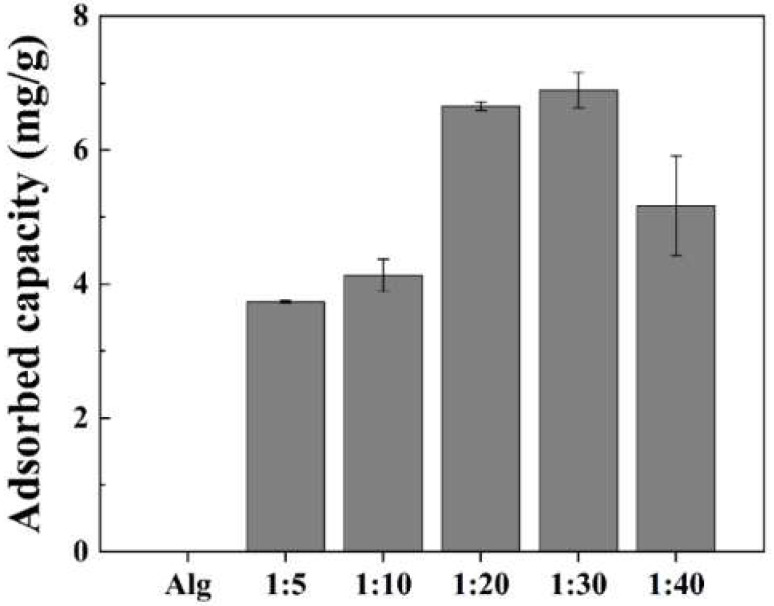
Iodide adsorption capacity of parent alginate (Alg) and beads prepared with different Alg and bismuth oxide (BO) weight ratios (initial iodide concentration: 20 mg/L, initial adsorbent concentration: 1 g/L, and contact time: 24 h).

The iodide adsorption capacity of the prepared Alg beads with/without BO was evaluated through simple batch adsorption experiments (Figure 1). The Alg beads without BO exhibited 0% iodide adsorption capacity even after 24 h. When BO was introduced, the iodide adsorption capacity gradually increased with the weight ratio (from 3.7 mg/g (1:5) to 6.9 mg/g (1:30)) after 24 h. However, when the weight ratio was increased to 1:40, the adsorption capacity decreased by approximately 25%. These results were attributable to the aggregation of BO particles when the amount of BO was increased in the Alg matrix for granulation [36,37]. Considering these preliminary iodide adsorption results for different ratios of Alg and prepared BO, Alg–BO with a weight ratio of 1:20 was selected for further study.

### 2.2. Characterization of Prepared Alg–BO

**Figure 2:**
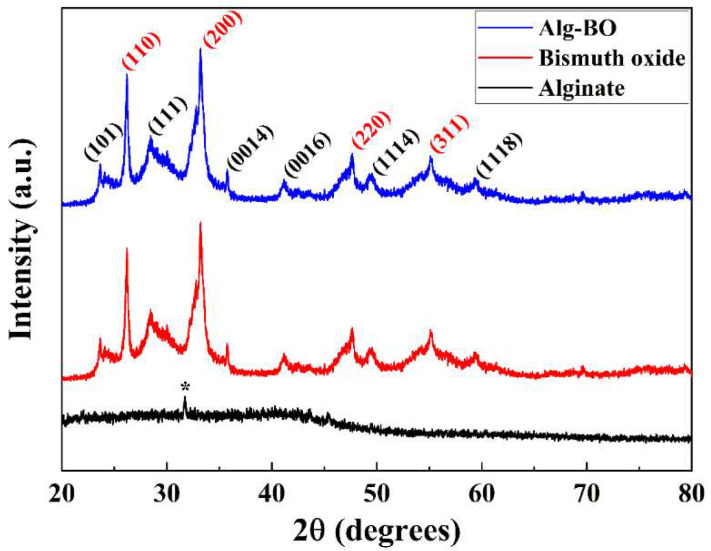
Powder X-ray diffraction patterns of Alg, BO, and Alg–BO (asterisk indicates (111) diffraction from Alg; black and red Miller indices indicate two bismuth oxide forms: Bi_2_O_2.33_ and γ-Bi_2_O_3_, respectively).

The Alg–BO (1:20) selected in the previous analysis was characterized by PXRD, FT-IR, and SEM. The PXRD patterns of parent Alg exhibited small diffraction at 31.8°, corresponding to (111) diffraction (asterisk in Figure 2), and broad, amorphous diffraction in the range of 20°–45°, consistent with the previously reported result [38]. The prepared BO consisted of two types of bismuth oxide forms: γ-Bi_2_O_3_ (PDF No. 00-027-0052) and Bi_2_O_2.33_ (PDF No. 00-027-0051). The major diffractions of the two types of BO—(110), (200) for γ-Bi_2_O_3_ and (101), (111) for Bi_2_O_2.33_—were well developed through the solvothermal preparation method. After the granulation of BO with Alg, the characteristic diffractions associated with the two BO forms were preserved, and the small diffractions from Alg disappeared owing to the small amount of Alg used (weight ratio of 1:20). The PXRD patterns indicated that the granulation of the prepared BO with Alg did not significantly influence the BO crystal structure. 

**Figure 3:**
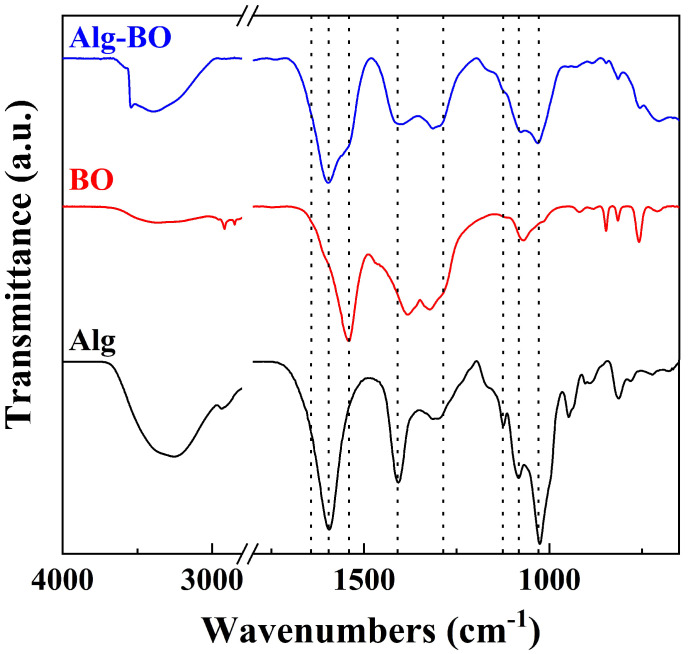
FT-IR spectra of Alg, BO, and Alg–BO (dotted lines: 1641, 1596, 1543, 1407, 1285, 1124, 1082, and 1025 cm^−1^).

Moreover, FT-IR spectroscopy was performed to identify the changes in the chemical properties of BO, Alg, and Alg–BO after granulation (Figure 3). In the FT-IR spectra of BO, characteristic stretching vibrations of O−H, C−H, and −CH_2_ were observed at approximately 3500–3200 cm^−1^ and 2800–3200 cm^−1^. The vibrations of (CH_2_)_n_, C−O, and C−O−C groups appeared at 700–1000 and 1100–1200 cm^−1^. The peaks at 1285 and 1641 cm^−1^ were attributable to the –COOH and C=O ester functional groups, respectively. The vibrations at 700–650 cm^−1^ originated from the metal-oxygen (Bi–O) vibrations. All vibrations attributable to BO were consistent with those observed in previous studies in which BO was prepared using the solvothermal method with an organic solvent such as ethanol or ethylene glycol [39,40]. Moreover, the intense vibration at 1543 cm^−1^ was attributable to the NO_3_ group, which indicated the existence of NO_3_ functional groups on the BO surface [39,41]. The spectra of the sodium alginate powder exhibited characteristic asymmetric and symmetric stretching vibrations of the carboxylate group (COO^−^) in Alg at 1407 and 1596 cm^−1^, respectively. The Alg–BO prepared by polymerization of Alg with CaCl_2_ exhibited characteristic vibrations attributable to both BO and sodium alginate [33]. Moreover, the C=O vibrations resulting from the ionic bonding between calcium ions and Alg was observed at approximately 1641 cm^−1^ as a shoulder, owing to the intense symmetric stretching vibrations of the carboxylate groups [42]. According to the PXRD and FT-IR results, BO was successfully incorporated with Alg by the polymerization and appeared in a bead form.

**Figure 4:**
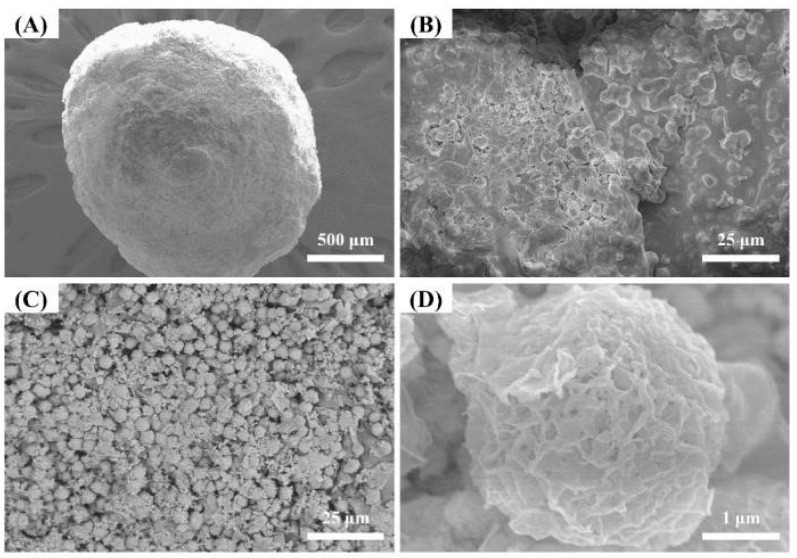
SEM images of the (**A**,**B**) surface and (**C**,**D**) cross-section of prepared Alg–BO.

To investigate the morphology of Alg–BO, SEM images of the surface and cross-section were obtained (Figure 4). As shown in Figure 4A,B, Alg–BO appeared as a spherical bead with a diameter of approximately 0.2–0.3 mm. The Alg–BO surface was smooth with BO particles (approximately 2–4 μm) aggregated with polymeric alginate. According to the cross-sectional images (Figure 4C,D), spherical BO particles (2–4 μm) were packed homogeneously in the Alg–BO, with irregular nanoparticles sized tens to hundreds of nanometers. The aggregates of rod or plate-like nanoparticles in the intraparticle pores likely enhanced the iodide adsorption. 

**Figure 5:**
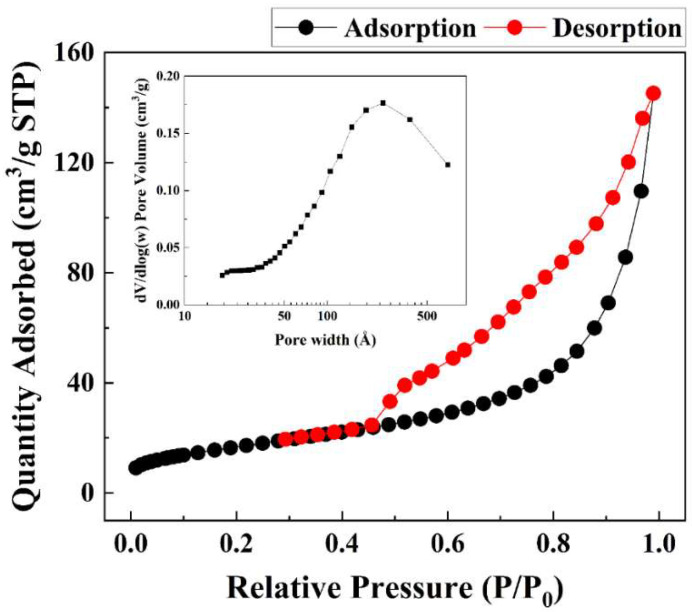
Nitrogen adsorption–desorption hysteresis loop of Alg–BO (inset graph shows the pore volume distribution).

According to the N_2_ adsorption–desorption hysteresis loop of Alg–BO (Figure 5), it can be classified as an H3 hysteresis loop based on the IUPAC classification [43]. This H3 hysteresis loop is known to be attributed to aggregates or agglomerates of particles with a nonuniform size and/or shape. This result corresponds quite well to the SEM images. The obtained specific surface area (SSA) was determined as 61.15 m^2^/g. This value shows an 18-44-times-higher SSA value than the previously reported SSA of bismuth oxide powder [44]. The increment in SSA might be attributed to Alg polymer, which makes it possible to form intraparticle pores between BO particles. In addition, the detailed pore width and pore volume calculated by the BJH method showed that the pores of Alg–BO were distributed in the range of 50 Å to 500 Å and mainly formed around 250 Å. 

### 2.3. Iodide Adsorption Performance of Alg–BO

#### 2.3.1. Adsorption Kinetics

**Figure 6:**
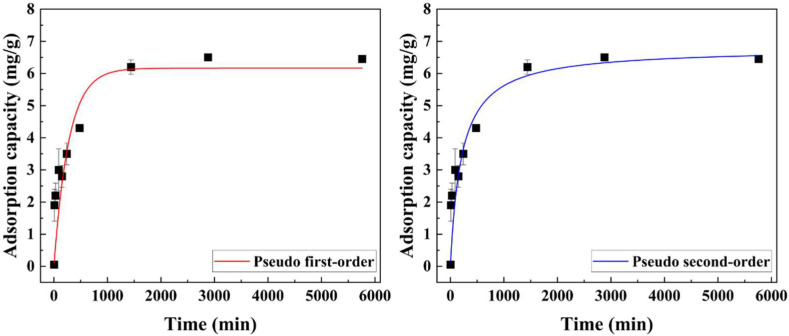
Iodide adsorption kinetics of Alg–BO and results of nonlinear fitting using pseudo-first- (red line) and pseudo-second-order (blue line) models. Initial iodide concentration = 20 mg/L; adsorbent concentration ≈ 1.4 g/L; contact time = 5–5760 min.

The iodide adsorption efficacy as a function of the contact time (5–5760 min) was evaluated with the initial iodide concentration being 20 mg/L. As shown in Figure 6, the prepared Alg–BO gradually adsorbed iodide for 480 min, and the capacity plateaued after 1440 min. This sustained adsorption, unlike that of powdered BO, was attributable to the granulation with Alg, which covered the BO surface. The kinetic results were fitted using pseudo-first-order (Equation (5)) and second-order (Equation (6)) kinetic models. According to the R^2^ values obtained using the two kinetic models, Alg–BO followed the pseudo-second-order instead of the pseudo-first-order kinetic model. Moreover, the kinetic parameters indicated that the adsorption rate and capacity of Alg–BO were 0.0007 g/mg·min and 6.792 mg/g, respectively.

The intraparticle diffusion model was applied to clarify the adsorption mechanism between iodide and Alg–BO, and the results are summarized in Figure S2 and Table S1. The intraparticle diffusion model involves three steps: (i) film diffusion of the liquid adsorbate onto the adsorbent surface, (ii) diffusion of the surface adsorbate into pores, and (iii) adsorption onto the inner surfaces of the pores [45]. As shown in Figure S2, the adsorption kinetics corresponded to intraparticle diffusion during 5760 min. The Alg–BO exhibited gradual adsorption through boundary layer diffusion during 480 min and reached the plateau from 1440 min to 5760 min, indicating intraparticle diffusion. This result implies that iodide ions steadily diffused into the intraparticle space of the Alg–BO beads for 5760 min. These results were consistent with those derived from the cross-section SEM images (Figure 4C,D), in which intraparticle pores were observed between spherical BO particle aggregates. 

#### 2.3.2. Adsorption Isotherms

**Figure 7:**
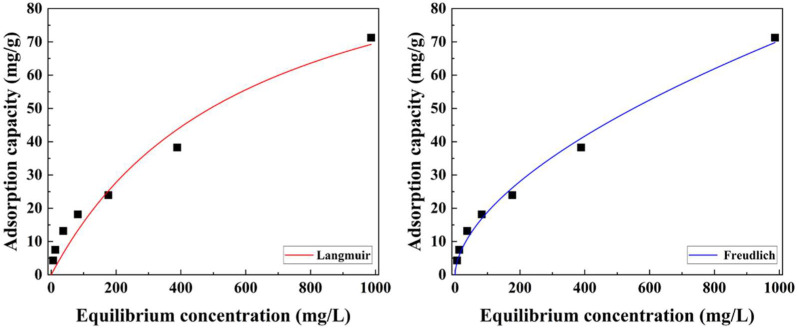
Iodide adsorption isotherm of Alg–BO and results of nonlinear fitting using Langmuir (red line) and Freundlich (blue line) models. Initial iodide concentration = 10–1000 mg/L; adsorbent concentration = 1 g/L; contact time = 24 h.

To evaluate the iodide adsorption capacity of the prepared Alg–BO, iodide adsorption isotherm experiments were carried out with iodide solutions having various initial concentrations (10, 20, 50, 100, 200, and 1000 mg/L) (Figure 7). The obtained results were analyzed using Langmuir (Equation (3)) and Freundlich (Equation (4)) isotherm models (Figure 7 and Table 1). As summarized in Table 1, the *q*_m_ for the Langmuir isotherm model was 111.8 mg/g, with high correlation coefficients (R^2^ values; 0.9561). It is worth noting that this *q*_m_ was approximately 9.0 times higher than the reported powdered bismuth oxide and 510 times larger than that for the polyacrylonitrile encapsulated bismuth oxyhydroxide nanocomposite, as summarized in Table 2. Moreover, the separation factor (*R_L_*; dimensionless constant) was calculated as the following equation [46]:

(1) $$R_{L}=\frac{1}{\left(1+a_{L}C_{0}\right)}$$

where *C*_0_ is the highest initial adsorbate concentration (mg/L) and *a*_L_ is the Langmuir constant (L/mg). The calculated *R_L_* indicated the adsorption isotherm and could be interpreted as unfavorable (*R_L_* > 1), linear (*R_L_* = 1), favorable (0 < *R_L_* < 1), or irreversible (*R_L_* = 0). The *R*_L_ of Alg–BO was 0.385, implying that the iodide adsorption on Alg–BO was favorable. Additionally, the Freundlich isotherm model was used to interpret the adsorption data considering a heterogeneous adsorption system. According to the fitting parameters and *n* value (1.750), Alg–BO represented a favorable iodide adsorbent. The R^2^ value for the Freundlich model was higher than that of the Langmuir model (0.9921). Therefore, the iodide adsorption by Alg–BO has been noted to correspond to monolayer iodide adsorption through the chemical interaction and precipitation between bismuth and iodide [47,48], followed by physical multilayer adsorption at a very high concentration of iodide in solution.

The obtained q_m_ value was compared to the reported value in the literature in Table 2. For the powder-type adsorbents, the copper-based adsorbents showed ≤2 mg/g of adsorption capacity, while zeolite and LDH showed ≤10 mg/g of adsorption capacity with a low selectivity toward iodide. The adsorption capacity could be enhanced by combining elemental silver or elemental Cu (22.9–25.4 mg/g). On the other hand, bismuth oxide (BO), which can be prepared in a simple procedure, showed 12.3–100 mg/g of iodide adsorption capacity. 

For the structured BO, polyacrylonitrile-based BO beads showed a very low iodide adsorption capacity due to their low BO content and low tested initial concentration. Even though the reaction condition was different, our result using Alg–BO showed that 111.8 mg/g of iodide adsorption capacity was one of the best results among bead-type adsorbents. This enhanced iodide adsorption capacity might be due to the 18-44 times larger SSA of Alg–BO than the SSA of powdered BO, which makes it possible to form intraparticle pores as a result of granulation with Alg [44]. Of course, several articles present higher adsorption performances using cellulose nanofiber and graphene, but our work still presents a meaningful result with a comparably high adsorption performance using a simple preparation method based on natural polymer, alginate.

#### 2.3.3. Effect of pH

**Figure 8:**
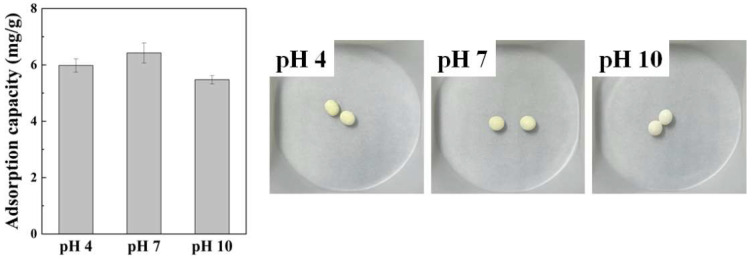
Iodide adsorption capacity as a function of initial pH of iodide solution (**left**) and photographs of Alg–BO after iodide adsorption (**right**). Initial iodide concentration = 20 mg/L; initial pH 4, 7, and 10; adsorbent concentration = 1 g/L; contact time = 24 h.

The iodide adsorption capacity as a function of initial pH was evaluated by simple batch adsorption experiments (Figure 8). At pH 4, the iodide adsorption capacity of Alg–BO exhibited a statically similar iodide adsorption capacity based on a t-test with a 95% confidence level. However, when the initial pH was increased to 10, the iodide adsorption capacity decreased by around 15%, from 6.42 mg/g to 5.47 mg/g. This decrement in iodide adsorption capacity at higher pH (pH 10) was attributed to the surface charge of bismuth oxide in Alg–BO. The point of zero charge (pH_pzc_) for BO was around pH 9.4, meaning that BO’s surface charge was shifted from positive to negative [62] at pH 10. Due to the negatively charged BO in Alg–BO, the adsorption of iodide could be interrupted by charge–charge repulsion, and it might lead to a decrement in iodide adsorption capacity.

### 2.4. Characterization of Alg–BO after Iodide Adsorption

The crystal structure and morphological changes of Alg–BO after iodide adsorption were investigated with PXRD and EDS-mapping-assisted SEM. In the PXRD patterns (Figure S3), characteristic diffractions attributable to two different types of BO (Bi_2_O_2.33_ and γ-Bi_2_O_3_) were observed. The relative intensity of diffractions slightly decreased (approximately 20%), but the crystal structure of Alg–BO was preserved even after 24 h. 

**Figure 9:**
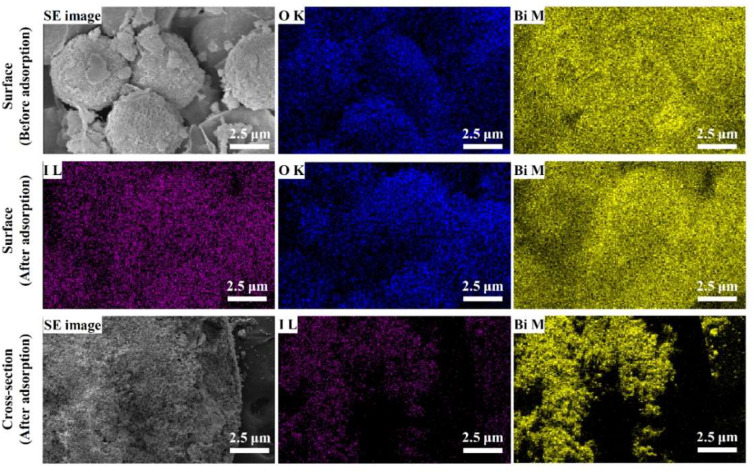
Element mapping images obtained by scanning electron microscopy of Alg–BO before and after iodide adsorption (blue: oxygen, yellow: bismuth, and magenta: iodide).

To visualize the distribution of the adsorbed iodide ion in Alg–BO, EDS-mapping-assisted SEM was performed (Figure 9). Before iodide adsorption, bismuth (yellow dot) and oxygen (blue dot) were homogeneously distributed on the BO particles, as observed in the SEM images. After exposure to the iodide solution, iodide ions (magenta dots) appeared not only on the surface but also in the cross-section. On the surface of Alg–BO, iodide ions appeared homogenously with bismuth and oxygen. Interestingly, the cross-sectional EDS mapping images indicated that the iodide was distributed only with bismuth. According to the EDS images, the adsorbed iodide ions interacted with BO in Alg–BO. In addition, from the FT-IR spectra of Alg–BO after iodide adsorption, the characteristic vibrations at 1641, 1596, 1543, 1407, 1285, 1124, 1082, and 1025 cm^−1^ attributed by alginate and BO were well-maintained after iodide adsorption (Figure S4). From the FT-IR spectra, iodide adsorption did not affect the chemical properties of Alg-BO. 

**Figure 10:**
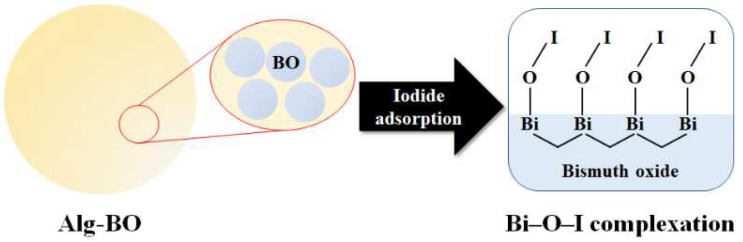
Schematic adsorption mechanism between Alg–BO and iodide.

Based on the previous study, bismuth oxide can form Bi–O–I bonding directly through chemisorption via Bi–O–I complexation (Figure 10) [48,59,63]. The chemisorption mechanism could lead to less desorption of bound iodide from the adsorbent; therefore, it is promising for handling radioactive contaminants.

Furthermore, the bismuth ion concentration in the supernatant was quantified to evaluate the stability of Alg–BO. The amount of dissolved bismuth ions was 2.6 μg/L, which was significantly low, indicating that the BO in Alg–BO was highly stable in the iodide solution for 24 h. Therefore, according to the iodide adsorption and characterization results, Alg–BO represents a promising iodide adsorbent, which has a controllable size and contents, can be easily managed, and can, thus, be applied in various fields. 

## 3. Materials and Methods

### 3.1. Materials

Bismuth (III) nitrate pentahydrate (Bi(NO_3_)_3_·5H_2_O; 98%), potassium iodide (KI; 99.5%), sodium hydroxide (NaOH; 97%), hydrochloric acid (HCl; 35%), and ethanol (94.5%) were purchased from Samchun Chemicals Co., Ltd. (Seoul, Korea). Sodium alginate was obtained from Junsei Chemical Co., Ltd. (Tokyo, Japan). CaCl_2_ was obtained from Dongyang Chemical Co., Ltd. (Yeongam-gun, Korea). Ethylene glycol (C_2_H_4_(OH)_2_; 99.8%) was purchased from Sigma-Aldrich Co., LLC (St. Louis, MO, USA). All chemicals were used without purification. Ultrapure water (deionized water; DI) was produced using a water purification system (Synergy^®^, Merck, Kenilworth, NJ, USA). 

### 3.2. Synthesis of Bismuth Oxide (BO)

To prepare BO, 0.97 g of bismuth (III) nitrate pentahydrate, ethanol (34 mL), and ethylene glycol (17 mL) (ethanol: ethylene glycol = 2:1 *v*/*v*%) were added to a 100 mL glass beaker and stirred with a magnetic stirrer for approximately 30 min until the bismuth nitrate completely dissolved. The prepared solution was transferred to a stainless-steel autoclave with a Teflon liner and hydrothermally treated in an oven at 160 °C for 10 h. The product (50 mL) was collected by centrifugation (6000 rpm for 5 min) in a conical tube. The obtained slurry was washed four times with a mixed solution of DI water and ethanol (1:1 *v*/*v*%) and dried in an oven at 60 °C. 

### 3.3. Granulation of Bismuth Oxide by Alginate (Alg–BO)

BO was granulated by dropping the Alg/BO mixed slurry into a CaCl_2_ solution, as described in our previous work [33]. First, the sodium alginate was dissolved in DI (10 mg/mL) by stirring for over 30 min with a mechanical stirrer. Subsequently, powdered BO was added to the Alg solution, with five weight ratios of Alg to BO considered (1:5, 1:10, 1:20, 1:30, and 1:40). This suspension was stirred for 2 h, transferred (5 mL) to a syringe, and added to a 2 *w*/*v*% CaCl_2_ solution dropwise through a syringe pump (NE4000, NEW ERA; 1.5 mL/min) with vigorous stirring. The beads generated in the CaCl_2_ solution were stabilized by stirring for 30 min, washed with DI water, and stored in a conical tube with DI.

### 3.4. Characterization

The PXRD patterns were obtained in the range of 20° to 80°, using the Bruker DE/D8 Advance (Bruker AXS GmbH, Berlin, Germany) with a 5 mm air-scattering slit and 2.6 mm equatorial slit, in timestep increments of 3.9 °/min. The FT-IR attenuated total-reflectance (ATR) spectroscopy (Spectrum two, Perkin Elmer, UK) results for a dried bead were obtained in the range of 450–4000 cm^−1^ with eight scans and a resolution of 4 cm^−1^. The size and morphology of the Alg–BO were determined through high-resolution field emission SEM (HR-SEM) using a Hitachi SU8010 (Hitachi High-Technologies Corporation, Tokyo, Japan) assisted by EDS (X-Max, Horiba, Kyoto, Japan) along with a 10 kV accelerated electron beam and a working distance of 8 mm. To perform the SEM/EDS analysis, the prepared Alg–BO was lyophilized and attached to a piece of carbon tape. To obtain cross-section images, the lyophilized Alg–BO was sliced using a surgical knife. Subsequently, the sample surface was coated with a Pt/Pd layer (approximately 10 nm thickness) by using a high-resolution sputter coater. Inductively coupled plasma-mass spectrometry (ICP-MS; Agilent 7900, Agilent Technologies, Inc., CA, USA) was performed to quantify the BO released in the supernatant after iodide adsorption. The nitrogen adsorption–desorption isotherm hysteresis loop and Brunauer–Emmet–Teller (BET) surface area were obtained by a 3Flex physisorption analyzer (Micromeritics, Norcross, GA, USA). The average pore volume and width were determined using the Barrett–Joyner–Halenda (BJH) method.

### 3.5. Iodide Adsorption Experiments

#### 3.5.1. Optimization of Granulation Conditions for Alg–BO

The variation in the iodide adsorption efficacy with the Alg:BO ratio was determined through a simple batch test to optimize the granulation conditions for Alg–BO. Approximately 40 mg of Alg–BO (1:5, 1:10, 1:20, 1:30, and 1:40 of wt%) was added to a 40 mL potassium iodide solution (20 mg I/L) and continuously agitated using a vertical shaker for 24 h. The initial pH of the iodide solution was adjusted to 7.0 using HCl and NaOH. The supernatant was collected using a syringe filter (polyethersulfone (PES), 0.45 μm). The iodide concentration in the supernatant was quantified by ultraviolet (UV) absorbance at a wavelength of 225 nm using a UV–visible spectrometer (UV-vis spectrometer, Genesys 50, Thermo Fisher Scientific, USA). After the adsorption experiments, the amount of iodide ions adsorbed per weight of adsorbent *q*_e_ (mg/g) was determined using Equation (2).

(2) $$q_{e}\;\left(\frac{mg}{g}\right)=\frac{\left(C_{0}-C_{e}\right)}{\left(\frac{m}{V}\right)}\;$$

where *C*_0_ is the initial iodide concentration (mg/L), *C_e_* is the equilibrium concentration after adsorption (mg/L), *m* is the adsorbent weight (g), and *V* is the volume of the solution (L).

#### 3.5.2. Iodide Adsorption Isotherm and Kinetic Experiments

Iodide adsorption isotherm experiments were conducted with initial iodide concentrations of 10, 20, 50, 100, 200, 400, and 1000 mg/L (pH 7). Approximately 30 mg of Alg–BO was dispersed in 30 mL of each iodide solution (1 g/L) and continuously shaken using a vertical shaker for 24 h. The sample was collected and quantified, as described in Section 3.5.1. The obtained isotherm result was fitted using the Langmuir (Equation (3)) [64] and Freundlich (Equation (4)) [65] isotherm models.

(3) $$q_{e}=\frac{\left(q_{m}a_{L}C_{e}\right)}{\left(1+a_{L}C_{e}\right)}$$

(4) $$q_{e}=K_{F}\cdot C_{e}^{\left(\frac{1}{n}\right)}$$

where *q_e_* is the quantity of adsorbate adsorbed per unit weight of solid adsorbent, *q_m_* is the maximum sorption capacity of the adsorbent (mg/g), *C_e_* is the equilibrium concentration of the adsorbate in solution (mg/L), and *a_L_* (L/mg) is the Langmuir affinity constant. *K_F_* is the Freundlich constant indicating adsorption capacity, and *n* is the Freundlich constant related to the favorability of the adsorption process.

Moreover, iodide adsorption kinetic experiments were conducted using 20 mg/L iodide solutions (pH 7) and 1.4 g/L of adsorbent dose. The reaction vessel was closed and gently stirred using a magnetic stirrer at 25 °C. The supernatant was collected and filtrated through a 0.45 μm PES syringe filter at designed time points (5, 10, 30, 90, 150, 240, 480, 1440, 2880, and 5760 min). The obtained supernatant was analyzed through a UV–vis spectrometer. The kinetic results were analyzed using pseudo-first-order (Equation (5)) [66] and pseudo-second-order [67] kinetic models (Equation (6)). Moreover, the intraparticle diffusion kinetics model (Equation (7)) [68] was used to investigate the adsorption mechanisms.

(5) $$q_{t}=q_{e}\left(1-e^{-kt}\right)$$

(6) $$q_{t}=\frac{k_{2}q_{e}^{2}t}{1+k_{2}q_{e}^{t}}$$

(7) $$q_{t}=K_{id}t^{\frac{1}{2}}+c$$

where *q_t_* is the adsorbed amount at time *t* (mg/g), *q_e_* is the equilibrium concentration (mg/g), *k* is the first-order rate constant (1/min), and *k*_2_ is the second-order rate constant (g/mg⋅min). Moreover, *K_id_* (mg/g∙min^1/2^) is the intraparticle rate constant, and c (mg/g) is the thickness of the boundary layer formed in the first interval.

#### 3.5.3. Effect of pH

To evaluate the effect of initial iodide solution pH, the pH of 20 mg/L of iodide solution was adjusted to pH 4 and 10 by HCl and NaOH, respectively. Around 30 mg of Alg–BO was placed into 30 mL of pH-adjusted iodide solution (1 g/L of adsorbent dose) and continuously agitated using a vertical shaker for 24 h. The sample was collected and quantified, as described in Section 3.5.1.

## 4. Conclusions

The BO was successfully granulated with Alg. The optimal condition for the granulation was determined considering different weight ratios of BO to Alg (1:5–1:40) in batch iodide adsorption experiments. The weight ratio of 1:20 wt% was selected as the optimal condition. According to the characterization results obtained through PXRD, FT-IR, and SEM analyses, BO appeared in two forms: Bi_2_O_2.33_ and γ-Bi_2_O_3,_ and was successfully granulated with Alg, yielding spherical beams with a diameter of 3 mm. According to the cross-sectional SEM images, irregular nanoparticles sized tens to hundreds of nanometers were packed into a few millimeters of the granulated adsorbent. The intraparticle pores in the granule could enhance the iodide adsorption. The iodide adsorption capacity of Alg–BO gradually increased and did not reach a plateau even at an initial iodide concentration of 1000 mg/L. Moreover, the calculated *q_m_* was 111.8 mg/g. According to the isotherm model analysis, iodide adsorption occurred as monolayer adsorption through the chemical interaction and precipitation between bismuth and iodide, followed by physical multilayer adsorption at a very high concentration of iodide in solution. Furthermore, the iodide adsorption as a function of contact time was analyzed by fitting with the intraparticle diffusion model through boundary layer diffusion during 480 min, reaching the plateau from 1440 min to 5760 min by intraparticle diffusion. EDS mapping images of the surface and cross-section after iodide adsorption indicated that the adsorbed iodide interacted with BO in Alg–BO through Bi–O–I complexation. This research shows that Alg–BO is a promising iodide adsorbent with a high absorption capacity, stability, and convenience, and it can help prevent secondary pollution.

## Figures and Tables

**Table 1:** Isotherm fitting and kinetic fitting parameters of iodide adsorption by Alg–BO.

	Iodide isotherm models					
Alg–BO	Langmuir			Freundlich		
	*q*_m_ (mg/g)	*a*_L_ (L/mg)	R^2^	*K*_F_	*n*	R^2^
	111.8	0.0016	0.9561	1.358	1.750	0.9921
	Iodide kinetic models					
	Pseudo-first-order equation			Pseudo-second-order equation		
	*q*_e_ (mg/g)	*k* (min^−1^)	R^2^	*q*_e_ (mg/g)	*k*_2_ (g/mg·min)	R^2^
	6.166	0.00358	0.8743	6.792	0.0007	0.9048

**Table 2:** Summarized iodine species adsorption capacity of various adsorbents.

Material	Sample Type	Iodine Species	Adsorption Capacity (mg/g)	Initial Iodide Concentration (mg/L)	Sample Dosing(g/L)	Contact Time	Ref
Activated bismuth oxide	Powder	I^−^	100	200	1.0	4 h	[49]
Bismuth oxide	Powder	I^−^	12.3	200	1.0	4 h	[49]
Cu_2_O	Powder	I^−^	0.3	13	50	5 d	[50]
Cu_2_S	Powder	I^−^	2.54	13	20	8 d	[51]
Mg-Al (NO_3_) LDH	Powder	I^−^	10.1	342.33	20	4 h	[52]
Modified zeolite	Powder	I^−^	3.6	10–500	10	24 h	[53]
Ag/Cu_2_O	Powder	I^−^	25.4	2.6–26	1.0	12 h	[54]
Cu/Cu_2_O	Powder	I^−^	22.9	2.6–39	1.0	12 h	[55]
silver nanoparticles-impregnated zeolite	Powder	I^−^	19.54–20.44	75–450	5.0	900 min	[56]
Polyacrylonitrile-bismuth oxyhydroxide	Bead	IO_3_^−^	0.216	1.0	5.0	24 h	[57]
Polyacrylonitrile-bismuth subnitrate	Bead	IO_3_^−^	0.199	1.0	5.0	24 h	[58]
Cu/Cu_2_O-immobilized cellulosic filter	Filter	I^−^	10.32	1–25	2.0	15 h	[59]
3D Graphene-Formicary-like δ-Bi_2_O_3_ Aerogels	Filter	I^−^	259.08	13–130	1.0	12.5 min	[60]
Nano-cellulose hydrogel coated flexible titanate-bismuth oxide membrane	Filter	I^−^	225.9	500	-	360 min	[61]
Alg–BO	Bead	I^−^	111.8	10–1000	1.0	24 h	This study

## Data Availability

The data presented in this study are available on request from the corresponding author.

## Supplementary Materials

The following supporting information can be downloaded at: https://www.mdpi.com/article/10.3390/ijms232012225/s1.
