# Supplementary material for: Granulation of Bismuth Oxide by Alginate for Efficient Removal of Iodide in Water
Source: Int J Mol Sci. 2022 Oct 13;23(20):12225. doi: 10.3390/ijms232012225 (PMC9603213; doi:10.3390/ijms232012225)
Supplement: Supplementary file 1 [file ijms-23-12225-s001.zip › ijms-1898199-supplementary.pdf]

Supporting information

Granulation of Bismuth Oxide by Alginate for Efficient Removal of Iodide in Water

Tae-Hyun Kim [1], Chihyun Seo [1], Jaeyoung Seon [1,2], Anujin Battulga [1] and Yuhoon Hwang [1,*]

[1] Department of Environmental Engineering, Seoul National University of Science and Technology, Seoul 01811, Korea
[2] Water Quality Center, Chemicals & Environment Research Institute, Korea Testing & Research Institute, Gyeonggi-do 13810, Korea
* Correspondence: yhhwang@seoultech.ac.kr; Tel.: +82-2-970-6626; Fax: +82-2-971-5776

Table of contents

Figure S1. Photographs of prepared Alg-BO with different weight ratio of Alg and BO........1

Figure S2. Iodide adsorption kinetic results fitted using the intraparticle diffusion model......2

Table S1. Intraparticle diffusion model parameters of Alg-BO for iodide adsorption................3

Figure S3. Powder X-ray diffraction patterns of Alg-BO after iodide adsorption......................4

Figure S4. FT-IR spectra of Alg, BO, Alg–BO, and Alg–BO after iodide adsorption................5

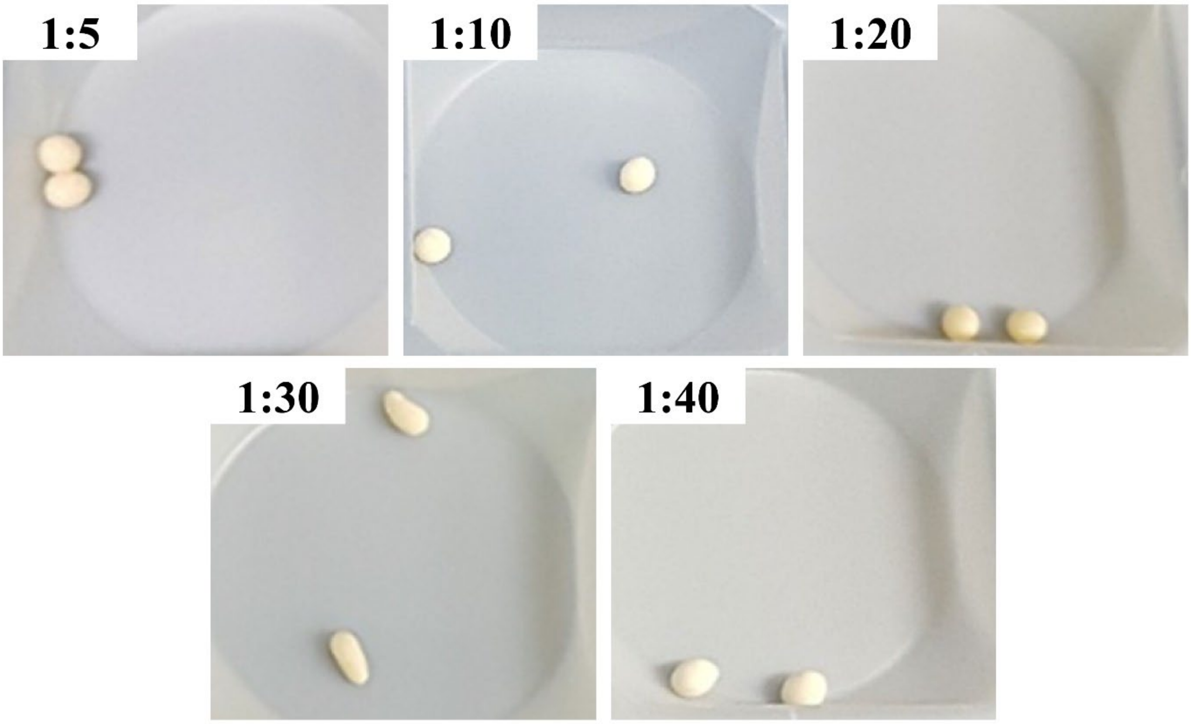

Figure S1. Photographs of prepared Alg–BO with different weight ratios of Alg and BO

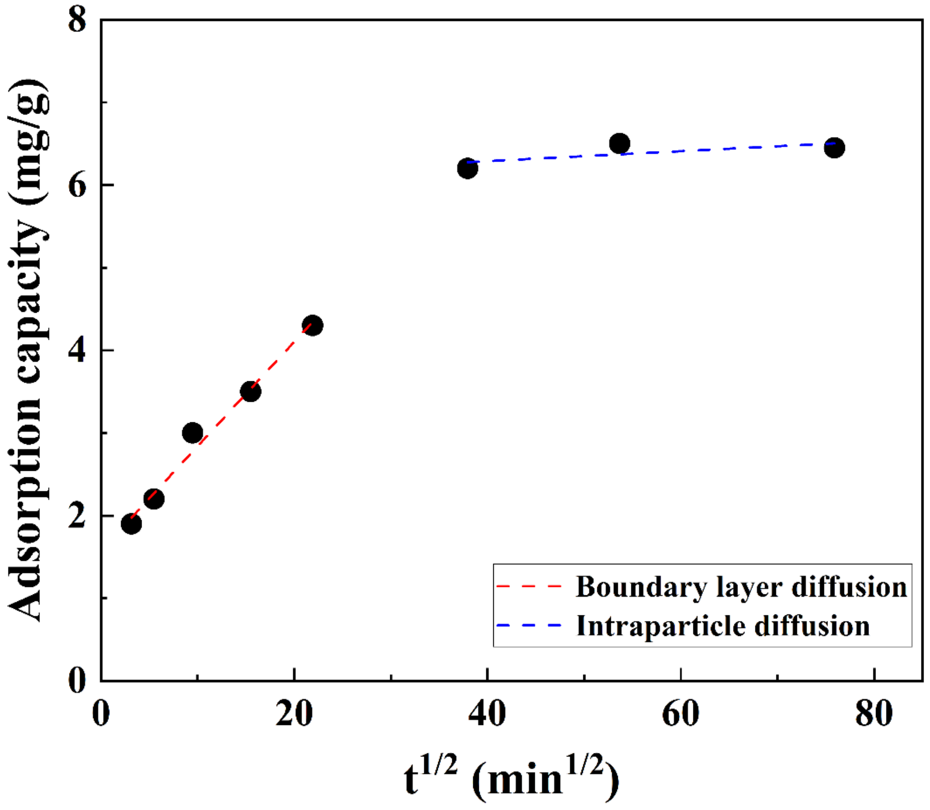

Figure S2. Iodide adsorption kinetic results fitted using the intraparticle diffusion model

Table S1. Intraparticle diffusion model parameters of Alg–BO for iodide adsorption

	First linear portion			Second linear portion		
**Alg–BO**	K_{id} (mg/g·min$^{1/2}$)	c	R^2	K_{id} (mg/g·min$^{1/2}$)	c	R^2
	0.1263	1.577	0.9831	0.006	0.3431	0.5069

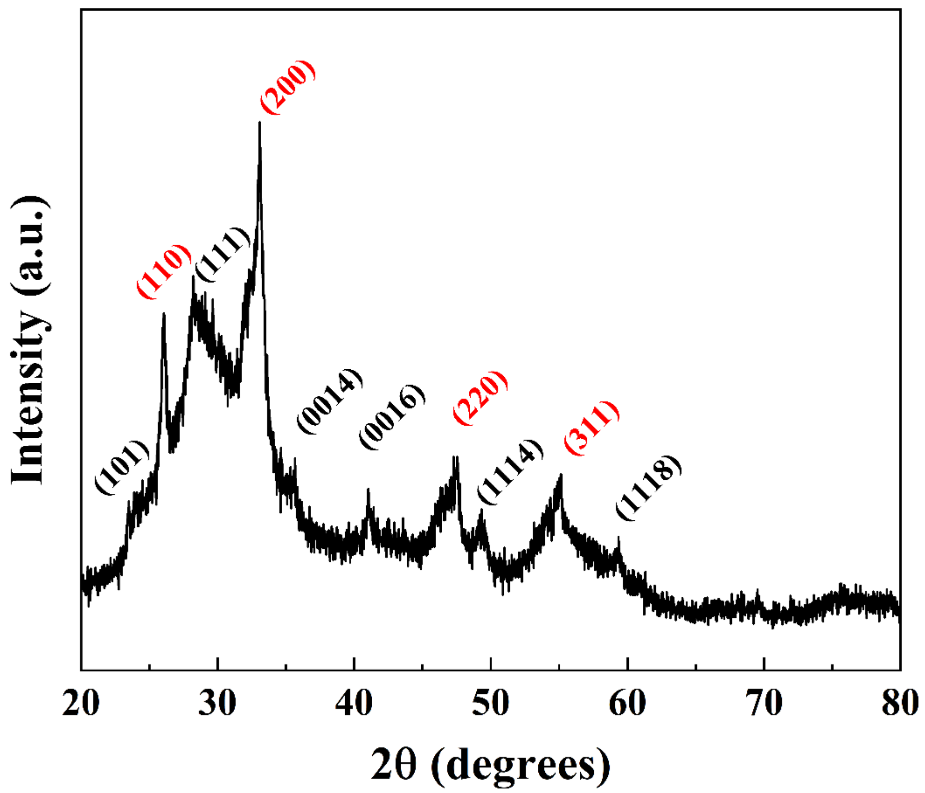

Figure S3. Powder X-ray diffraction patterns of Alg–BO after iodide adsorption (black and red Miller indices indicate two forms of bismuth oxide: $Bi_2O_{2.33}$ and γ-Bi_2O_3, respectively)

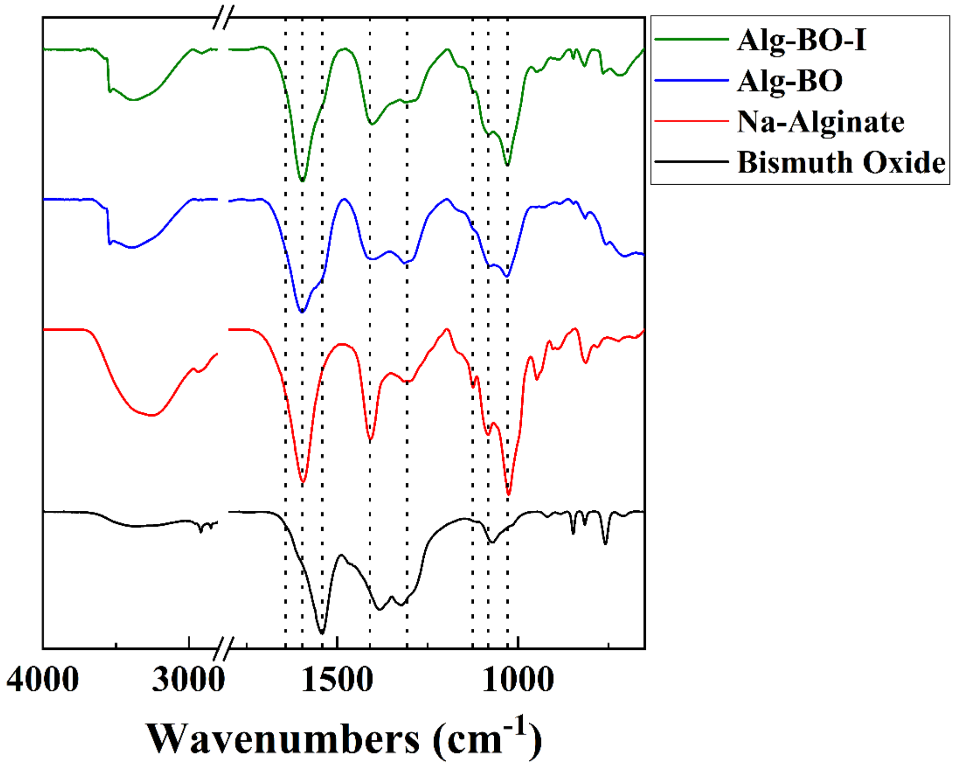

Figure S4. FT-IR spectra of Alg, BO, Alg–BO, and Alg–BO after iodide adsorption (dotted lines: 1641, 1596, 1543, 1407, 1285, 1124, 1082, and 1025 cm^{-1}).
